# Co-Designing a Consumer-Centred Educational Resource to Assist Australian Farmers with the Management of Persistent Pain

**DOI:** 10.3390/ijerph23081015

**Published:** 2026-08-03

**Authors:** Indika Koralegedera, Felicity A. Braithwaite, G. Lorimer Moseley, Gemma Skaczkowski, Kate M. Gunn

**Affiliations:** 1 Adelaide University Rural Health, College of Health, Adelaide University, Adelaide 5000, Australia; gemma.skaczkowski@adelaide.edu.au; 2 IIMPACT in Health, College of Health, Adelaide University, Kaurna Country, Adelaide 5000, Australia; felicity.braithwaite@adelaide.edu.au (F.A.B.); lorimer.moseley@adelaide.edu.au (G.L.M.); 3 Persistent Pain Research Group, Hopwood Centre for Neurobiology, Lifelong Health Theme, South Australia Health and Medical Research Institute (SAHMRI), Adelaide 5000, Australia

**Keywords:** pain management, farmer, co-design

## Abstract

**Highlights:**

**Public health relevance—How does this work relate to a public health issue?**

- Persistent pain is a significant burden affecting farmers physically, psychologically, socially and financially.
- Farmers respond best to health-related educational resources tailored to their specific preferences and needs.

**Public health significance—Why is this work of significance to public health?**

- Transferable lessons from the development of the first co-designed, evidence-based, farmer-friendly pain management education resource for Australian farmers are shared to inform the development of other health-related resources for this at-risk group.

**Public health implications—What are the key implications or messages for practitioners, policy makers and/or researchers in public health?**

- Pain management education resources are well-received when tailored to the target population in content, format, mode, language and aesthetics.
- Focusing on function rather than solely on pain, encouraging reflection and goal setting and ensuring that pain education is relevant across their pain journeys (at pain onset, the frustration stage, and the resignation stage), is important to Australian farmers.

**Abstract:**

Persistent pain is a major health issue that interferes with farmers’ ability to work more commonly than for the general working population. Farmers also like to self-manage pain but evidence-based pain management educational resources that are relevant to their way of life are limited. This study utilised a participatory co-design approach with farmers, pain clinicians and pain researchers to develop a tailored persistent pain management education resource for farmers. Key, transferable learnings include: the value of information provision in both printed and digital formats, the use of straightforward language, farmer-themed metaphors, imagery, humour and case studies/quotes, as well as reflection and goal-setting activities that focus on function. Co-designers highlighted the importance of materials being easily accessible, sharable and distributed by those who can demonstrate an understanding of farming, to reassure farmers of the resources’ relevance. Including information that is applicable across farmers’ typical pain journeys (pain onset, frustration and resignation stages as described in the paper) is another important consideration. Findings have informed the development of a pain management education resource that was considered relevant, relatable and accessible by co-designers. Lessons are applicable to the development of future health-related educational resources for this unique and at-risk group.

## 1. Introduction

Persistent pain is a major health issue affecting around 20–40% of the global population [1,2,3]. Farmers are particularly affected, reporting a higher overall prevalence of persistent pain ranging from 24–52% [4,5,6]. In Australia, the self-reported prevalence of persistent pain among farmers is lower than these global estimates, at 7.8%; however, these farmers also report greater interference of pain in their daily lives than the general working population in Australia (18.5% vs. 11.8%) [7]. This discrepancy suggests a possible tendency among Australian farmers to underreport their pain.

Farmers often perceive pain as a normal part of life [8,9], despite its impact on various aspects of their lives. Their identities are also thought to be characterised by stoicism and adherence to strong cultural beliefs, such as the value of toughness, masculinity, independence, and self-reliance [10,11], which may hinder their willingness to seek help for health issues [10,12]. Hull, et al. [13] summarise the barriers farmers face to accessing health care as a combination of (a) personal attitudes and beliefs, (b) health system factors and (c) farm-related factors. These barriers may lead to adverse health outcomes for farmers, including in the management of persistent pain [14].

There is a need to develop self-management approaches to improve health outcomes for farmers [15]. Existing research suggests that farmers often prefer to self-manage their health problems, without seeking professional support [16]. Further, many of their self-management practices do not align with evidence-based guidelines [17], such as the use of cannabinoids, massage, and Bowen therapy [9]. Best-practice guidelines emphasise that pain education is a crucial component in managing persistent pain [18]. Evidence-based pain education could be particularly beneficial for farmers by assisting them to overcome the aforementioned barriers to help-seeking and supporting them to self-manage their pain more effectively, particularly when healthcare support is not available [19]. Increased knowledge and understanding of pain and its management has been shown to consistently lead to better pain outcomes in other populations [20], by reducing pain intensity and improving function, self-efficacy and quality of life [21,22].

Farmers are also known to prefer health-related information that is ‘farmerised’ or targeted to their specific communication style, culture and needs [23]. However, globally, there are limited farmer-focused educational resources and initiatives that provide this. In the United States, the University of Maine Cooperative Extension [24] offers a brief factsheet for farmers focusing on pain triage and work assessment, while the Upper Midwest Agricultural Safety and Health Center [25] and the Ohio State University Extension [26] also provide information on back pain prevention and management for farmers. In Australia, Boughton and Fragar [27] have developed a fact sheet focused solely on back pain prevention and management for farmers. These resources offer relevant information for farmers; however, most focus mainly on preventing and managing back pain specifically, or ergonomic modifications and exercise, rather than self-management of persistent pain more generally and farmer-friendly pain education.

It should also be noted that in Germany, Braun et al. [28] have developed an online Acceptance and Commitment Therapy to reduce chronic pain disability among green workers (including farmers). In Thailand, personalised self-care programmes for rubber farmers [29] and behavioural modification programmes [30] have been tested, while in South Korea, a mobile-delivered self-exercise programme is available to female farmers [31]. Although these initiatives generally all show improved pain outcomes for study participants, they require ongoing investment into the delivery of professional support that accompanies them, which in turn limits their scalability and reach, and may not suit farmers who report a strong preference to self-manage their pain on their own.

To our knowledge, there are not currently any evidence-based pain education resources specifically designed to help Australian farmers understand and self-manage persistent pain, despite recent research highlighting the value of this [9]. To effectively develop a pain management resource tailored to farmers, it is essential to understand their perspectives on barriers, facilitators, and preferences [32]. Involving both farmers (the primary end-users) and clinicians with expertise in persistent pain management and experience treating farmers in the co-creation of educational resources is likely to increase face validity and acceptability of the resource in farming communities [33]. Health education campaigns that are not co-designed with targeted learners risk exacerbating health inequalities [34]. More broadly, Patient and Public Involvement (PPI) is widely recognised as crucial to reduce waste in health research by ensuring that outcomes are meaningful and acceptable to end-users [35,36]. Therefore, this study aimed to collaboratively develop a persistent pain management education resource for Australian farmers, in partnership with farmers and rural pain clinicians. More specifically, the study explores the following research questions: (a) What is the preferred content, mode, and format of a pain management resource for Australian farmers? (b) What elements should be included or excluded to maximise the chance that the resource is culturally appropriate, safe, acceptable, and effective for Australian farmers? (c) What are the potential barriers and facilitators influencing farmers’ utilisation of the resource, and what strategies can be implemented to address these barriers?

## 2. Materials and Methods

### 2.1. Ethical Approval

Ethical approval was obtained from the University of South Australia Human Research Ethics Committee (project number: 206146).

### 2.2. Protocol

This research protocol was submitted before data analysis and locked on the Open Science Framework (https://doi.org/10.17605/OSF.IO/5ZEUQ). All deviations from the original protocol are documented in this manuscript and detailed in Table S1 (Supplementary File). The study was reported in accordance with the Guidance for Reporting Involvement of Patients and the Public (GRIPP2) short form (see Table S6) [37].

### 2.3. Study Design

This study was informed by a participatory co-design approach. We based our framework on the conceptual model developed by Braithwaite et al. [38], which aimed to establish a collaborative partnership that valued both the processes (quality of participatory strategies) and the outcomes of the process (control over decision-making). Co-design participants were considered ‘co-researchers’, with their input taken as seriously as the rest of the research team’s input, whilst using participatory strategies to reduce perceived hierarchies and ensure genuine engagement as equal partners in the design process.

### 2.4. Co-Design Team

The co-design team consisted of farmers with lived experience of persistent pain, pain clinicians working in rural clinical practice, and the research team. Farmers contributed their lived experiences, cultural views and preferences, while clinicians provided clinical expertise in helping farmers manage their persistent pain. We ensured that each workshop included at least an equal number of farmers as pain clinicians, with the aim of creating an environment that reduced perceived hierarchical power dynamics and where farmers feel comfortable sharing their perspectives. This approach is a crucial step toward achieving meaningful collaborative outcomes, as disparities often exist between the expectations of rural communities and those of their clinicians [17]. The research team included pain researchers and rural health and farmer wellbeing researchers with backgrounds in physiotherapy and clinical psychology. They offered expertise in best-practice pain management and pain education, co-design, participatory methods, and rural-focused research. Farmers were offered compensation in the form of a $100 AUD Coles Group and Myer gift voucher. Clinicians were not offered compensation.

### 2.5. Sampling and Recruitment

The study was promoted through flyers, email, print, radio, and social media, with recruitment supported by personal, rural, professional, industry, and farming networks. A convenience sampling approach was used to recruit farmers and pain clinicians. Drawing on our expertise in co-design research and collaborations with farmers and rural pain clinicians, we chose to recruit participants for small-group workshops. A small group of participants helps create a friendly environment that encourages active engagement [39,40]. Based on our team’s co-design experience and guidance from the focus group literature [40], we intentionally aimed to recruit a small group of up to 10 farmers and 10 clinicians to facilitate in-depth interactions, equitable participation, and deep generative engagement. The priority of this study was iterative, collaborative knowledge generation rather than data saturation or information power. See Table 1 for eligibility criteria.

### 2.6. Co-Design Workshops

**Figure 1:**
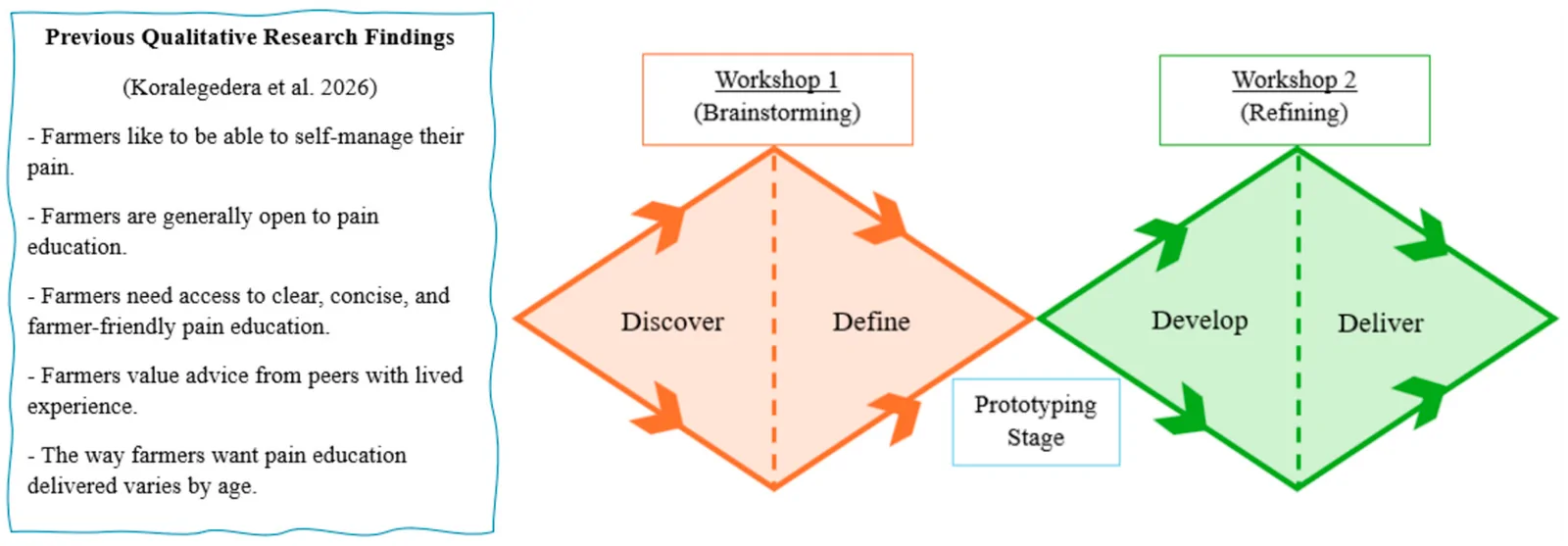
Double Diamond Process [9].

We conducted two stages of iterative co-design workshops: (1) the brainstorming stage and (2) the refining stage. These workshops aimed to understand farmers’ priorities regarding a pain management resource, brainstorm the format and mode of the resource, review the resource prototype, and refine the final version. Workshop content was informed by our previous qualitative work with farmers, who identified the need for a farmer-focused pain management resource and revealed farmer-specific requirements [9]. The research team developed the prototype resource content (prototyping stage) based on findings from the aforementioned study and the brainstorming stage, drawing upon the content expertise within the research team. Further, we hired an illustrator with experience in creating illustrations for farmer-focused resources for Australian farming communities. We adapted the Double Diamond design process developed by the British Design Council to inform our co-design methods, as detailed in Figure 1 below [41].

Before conducting the co-design workshops, we ran a pilot workshop with two farmers, representing both older and younger generations (aged 60 and 22), to ensure the relevance and acceptability of the workshop activities. Two members of the research team and one external observer also attended the pilot workshop (see Table S2 for the suggestions collected during the pilot workshop and adaptations to workshop activities based on these suggestions).

Video conferencing was employed as the mode for the co-design workshops due to its flexibility in spatial and temporal aspects. This platform is widely accepted in co-design contexts [42], including with farmers, and enabled participants from regional and rural areas across Australia to participate regardless of geographical constraints or accessibility challenges [43,44]. All workshops lasted approximately two hours. All workshops were facilitated by the lead investigator (IK), with one or two research team members (FAB and KMG). Discussions were facilitated using prompt questions, collaborative tasks, consensus methods such as voting, and case scenarios (see Table 2).

### 2.7. Data Analysis and Verification

Both co-researcher-led analysis during workshops and researcher-led analysis post-workshops were undertaken. For co-researcher-led analysis, the Miro online platform (RealTimeBoard, Inc., San Francisco, CA, USA; https://miro.com; accessed on 31 July 2026) was used in real time to map the pain journey of farmers. The Mentimeter online programme (Mentimeter AB, Stockholm, Sweden; https://www.mentimeter.com; accessed on 31 July 2026) was utilised to gain consensus for the participant-voting activities during the workshops (see Table 2 for workshop activities and questions). At the beginning of the refining workshops, participants were provided with summaries of the analysis from the earlier brainstorming workshops and verified how we had interpreted the earlier workshop data. For researcher-led analysis, inductive, iterative analysis methods were employed to examine the content of the workshop activities. Certain elements were systematically coded manually in Microsoft Excel (Microsoft 365, MSO version 2501, Microsoft Corporation, Redmond, WA, USA) into themes and sub-themes to inform the development of future components for the persistent pain management resource. All outcome-oriented workshop data were descriptively analysed by the lead investigator (IK). Two other researchers independently translated and verified workshop data concerning the pain mapping journey (FAB) and the rural farmer context (KMG). All members of the research team (IK, FAB, GLM, GS, and KMG) were involved in developing prototype content and refining the prototype, after discussing the discrepancies and reaching consensus.

## 3. Results

### 3.1. Co-Researchers (Participants)

Five farmers experiencing persistent pain and twelve rural pain clinicians expressed interest in participating in this study. Of these, four farmers (South Australia n = 2, Victoria n = 2) and four clinicians (South Australia n = 2, Victoria n = 1, New South Wales n = 1) indicated their willingness to commit to all stages of the study. However, attendance varied across different stages (see Table 3). These farmers commonly reported persistent pain, including back pain (n = 3), knee pain (n = 3), and upper-extremity pain (n = 2), as well as multiple injuries (n = 3) throughout their farming life. Three clinicians reported having a farming background.

### 3.2. Workshop Findings

At Workshop 1 (Brainstorming stage), we addressed all three research questions, placing emphasis on the format and modes to use in a pain education resource tailored to farmers.

#### 3.2.1. Preferred Content, Mode, and Format of the Pain Management Resource

Co-researchers reported that they would want a pain management education resource to manage their pain and seek comprehensive information on pain and effective pain management strategies, proactive measures to manage pain and workload, recognition of warning signs, understanding the psychological dimensions of pain, maintaining workplace safety, learning self-management techniques, and accessing available support resources. After a consensus-seeking activity, the co-researchers highlighted that the primary objectives of using a pain management education resource for farmers are to (1) **gain a comprehensive understanding of pain** and (2) **help them adopt practical strategies to manage their pain effectively**. These objectives were incorporated into the prototype through enhancing farmers’ understanding of pain and educating them on effective pain management strategies using established pain science education principles [45].

Co-researchers revealed that they would primarily seek self-management strategies in a pain management resource, including practical actions they can implement, and guidance on when and where to seek professional assistance. Given the diversity in the farming population (e.g., age), the co-researcher team emphasised that various types of information delivery formats and modes would also be required, including videos, podcasts, and documents. This information has been integrated into the prototype resource by providing a comprehensive list of reputable existing resources that are freely available and use a range of formats. Co-researchers suggested that farmers would **favour utilising both printed and digital formats** for the pain education resource. In line with our previous qualitative findings [9], co-researchers agreed that older-generation farmers tend to prefer printable materials, whereas younger farmers may favour online formats. However, they also raised that the accessibility of online formats can be influenced by factors such as internet connectivity and the size of downloadable files or web pages. In response to these considerations, the prototype resource was designed to be accessible through both online and printable downloadable formats.

#### 3.2.2. Elements to Be Included or Excluded to Ensure the Resource Is Culturally Appropriate, Safe, Acceptable, and Effective for Australian Farmers

The co-researchers emphasised that employing **clear and straightforward language, farmer-themed metaphors, imagery, humour and case studies/quotes** are effective strategies for disseminating educational information about pain management to farmers. The suggestions were integrated into the development of the prototype’s content, prioritising clarity and simplicity in language, supplemented by metaphors related to farming (e.g., a broken tractor oil gauge to convey the concept of an overprotective pain system).

Co-researchers suggested that general practitioners (GPs), physiotherapists, other farmer peers, and farming-related groups would be effective channels for conveying or distributing pain management educational resources to farmers. The importance of **anyone communicating this information having a clear understanding of farming was emphasised, to enable them to do this effectively and reassure other farmers of the relevance**. The prototype, therefore, included a statement that the resource was developed in partnership with farmers to enhance perceived credibility. Furthermore, they suggested that farmers would be most likely to access the resource if it was recommended by trusted peers, for example, other farmers or their spouse/partner.

At the beginning of the Workshop 2 (refining workshops), the chief investigator (IK) presented the findings from the previous workshop and sought respondent validation for the translation of the data from that workshop. All co-researchers agreed with no corrections or suggestions offered. Then, we covered all three research questions, with a stronger focus on the acceptability of the prototype content. The initial impression of the co-researchers on the prototype pain education resource was that it was **relatable, simple, understandable for farmers, introductory, provided a great summary, used good analogies, was informative, and encouraging**. Additionally, they emphasised the importance of the **reflection and goal-setting activities** at the end of the resource.

#### 3.2.3. Barriers and Facilitators Influencing Farmers’ Utilisation of the Resource, and the Strategies That Can Be Implemented to Address These Barriers

The co-researchers indicated that farmers typically seek out pain management educational resources when medications fail to alleviate their pain. Further, they mentioned that **farmers are likely to have more favourable outcomes when these resources motivate them to set and monitor their goals, as well as to focus on function rather than solely on pain**. We therefore ensured that the prototype content was crafted to effectively motivate farmers to actively participate in pain management strategies by incorporating specific self-directed activities such as goal-setting and reflection. Co-researchers expressed that for broader acceptance of the resource, it should make a material difference to farmers’ capacity to work. Then farmers would be highly likely to recommend the resource to their peers. Additionally, the resource should be **easily accessible and simple to share, to help farmers recommend it to others**.

#### 3.2.4. Mapping Farmers’ Pain Journeys

**Figure 2:**
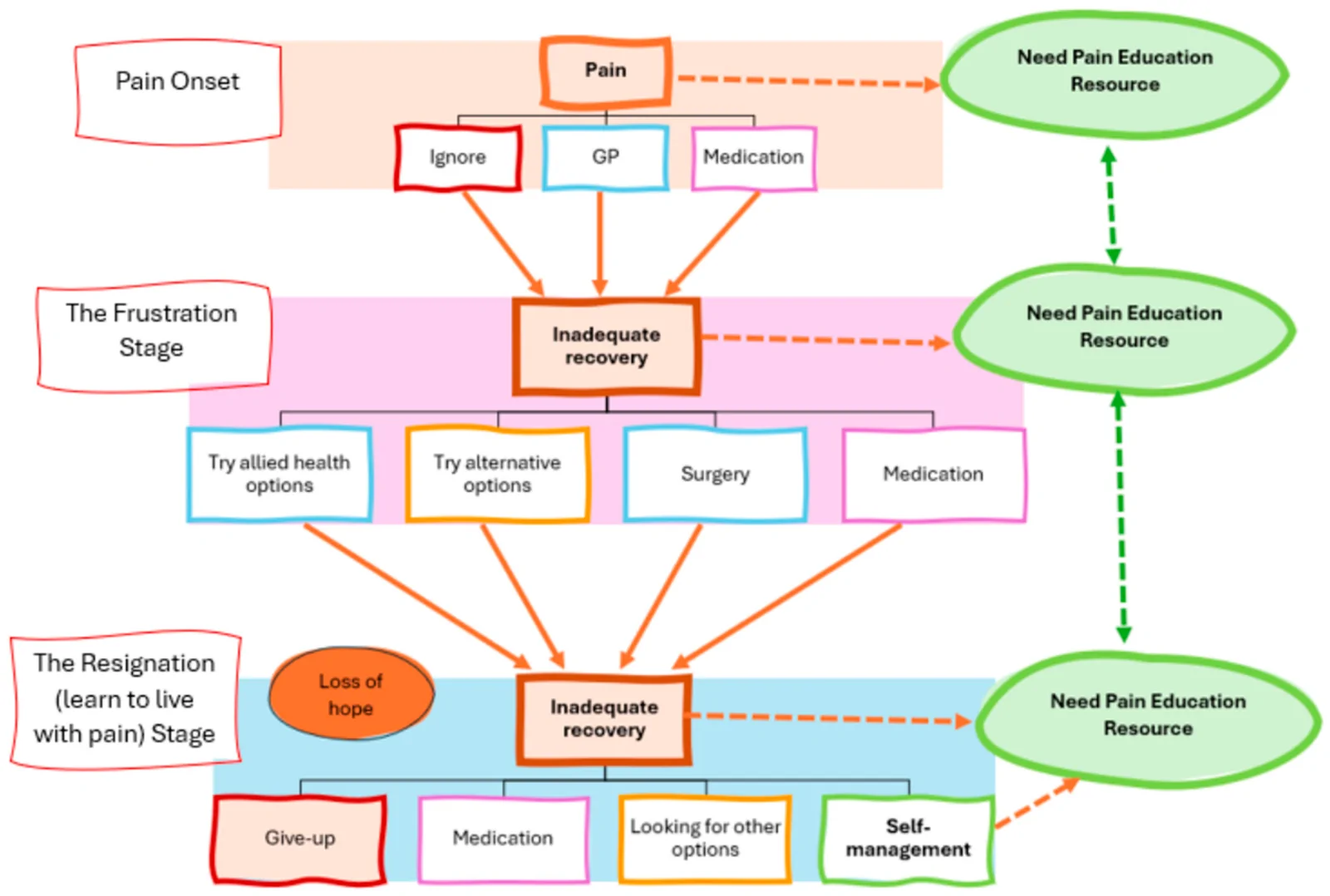
Typical pain journey of farmers.

We used pain journey mapping to identify farmers’ needs and the critical timepoints when they need a pain education resource [46], as detailed in Figure 2 below. This conceptual model represents participants’ shared experiences and perceptions of farmers’ pain trajectories and arose solely during the analysis of the pain mapping journey activity; therefore, it does not represent an empirically validated model.

This exercise revealed that farmers typically experience a relatively consistent linear progression through three stages of a pain journey, as shown in Figure 2. In the **first stage, pain onset, farmers generally adopt three strategies: (1) ignoring the pain; (2) consulting their general practitioner (GP); and/or (3) using pain-relieving medications**. However, the majority do not achieve adequate recovery at this stage, leading to feelings of hopelessness and progression to the second stage—the frustration stage. In this **frustration stage, farmers often then attempt four approaches: (1) seeking support from allied health professionals; (2) exploring alternative therapies; (3) undergoing surgical interventions; and/or (4) continuing medication use**. Despite these efforts, most farmers still perceive their pain management as insufficient, resulting in further despair and abandonment of pain management efforts, which marks the final stage of their pain journey—the resignation stage (learning to live with it). At the **resignation stage, farmers tend to choose among three approaches: (1) continue relying on medications; (2) explore new or previously untried interventions; or (3) attempt to self-manage their pain**.

Co-researchers emphasised the importance of **including information relevant to each of these three critical time points** in a pain education resource for farmers at each stage of their pain journey, especially at the first stage of pain onset. They further emphasised the importance of implementing **pain prevention programmes early in farmers’ careers**. Unfortunately, this prevention aspect could not specifically be incorporated into the prototype as it does not align with the resource’s primary objective to serve as a persistent pain *management* resource. However, the resulting resource could certainly be promoted to and adopted by those working with early-career farmers (e.g., at agricultural colleges), to make them aware of relevant approaches should pain become an issue for them, at any point in their working lives.

#### 3.2.5. Other Suggestions and Feedback on the Prototype

During Workshop stage 2, the co-researchers proposed a series of minor amendments to the prototype. They accepted the prototype with minor revisions, without needing to hold another workshop to reach consensus (See Table S4 for the complete list of actions and suggestions, and Table S5 for Example Quotes). See Table 4 for key suggestions and how the suggestions were incorporated into the prototype resource. These suggestions included **ensuring gender and age diversity in the illustrations**, emphasising the **importance of seeing a health care professional to rule out serious underlying health conditions** prior to engaging in self-management, and the value of the **inclusion of quotes in first person with context** (e.g., age).

**Figure 3:**
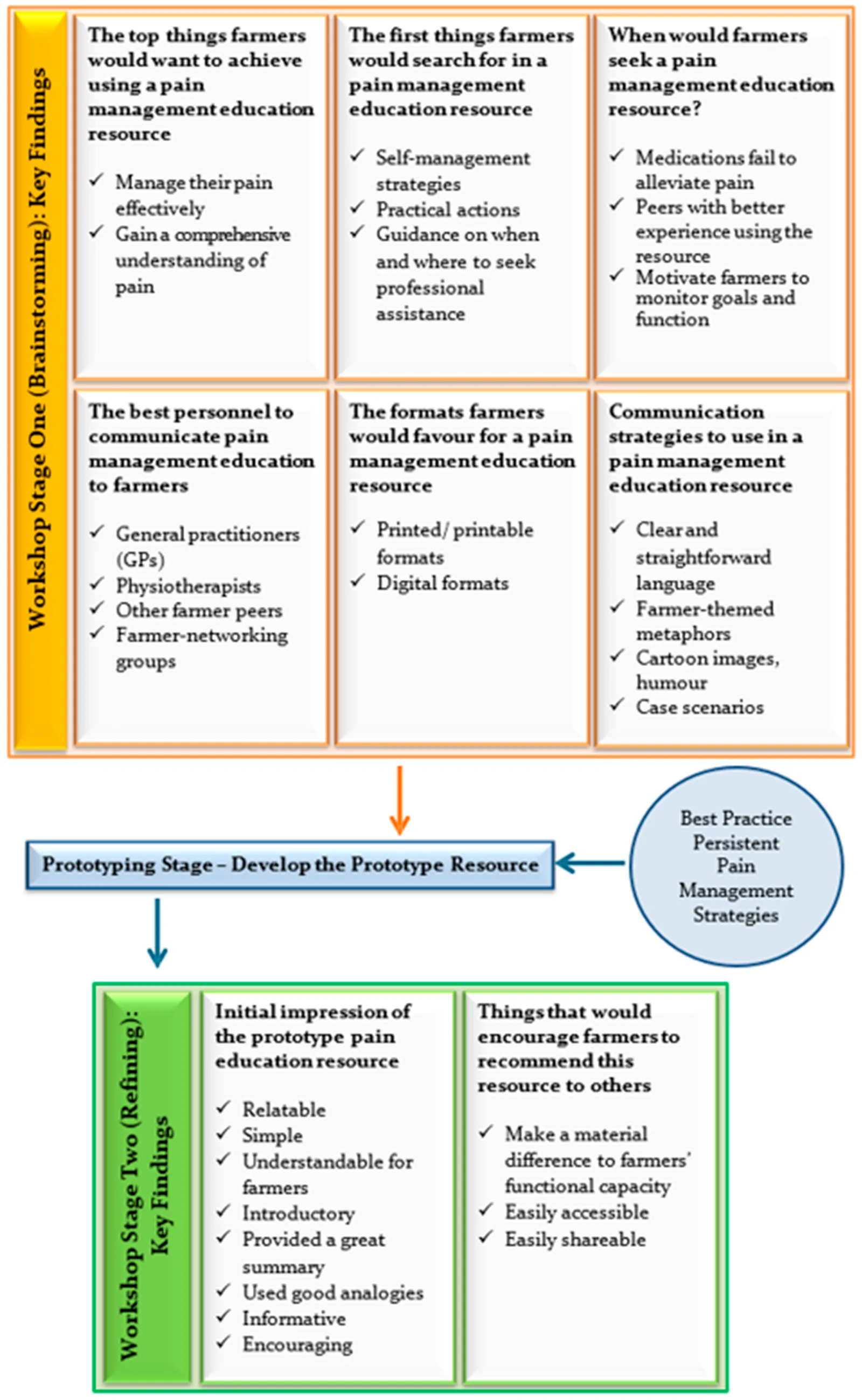
Summary of the key findings of the Workshop Stage 1 (brainstorming) and Workshop Stage 2 (refining).

The suggestions and feedback were incorporated, and the final prototype was updated following Workshop stage 2 (refining). See Figure 3 for the summary of the key findings of the Workshop stages 1 and 2. The finalised prototype can be accessed via: (https://ifarmwell.com.au/practical-tips-to-improve-your-wellbeing/understanding-and-managing-chronic-pain; accessed on 31 July 2026).

## 4. Discussion

The purpose of this work was to co-design an educational resource on persistent pain management, tailored for Australian farmers, in partnership with farmers and rural pain clinicians. The participatory methodology incorporated collaborative design-thinking activities that brought together diverse lived experiences and clinical and content expertise to ensure that the resource was relevant, relatable, and farmer-friendly, and comprised evidence-based pain management content that was both safe and suitable for implementation. Additionally, by revisiting farmers’ “pain journeys”, co-researchers in this study identified three critical time points at which a pain management resource would be best placed to support farmers—pain onset, the frustration stage, and the resignation stage—and the resource content was tailored to these time points accordingly. The main goals of the resource are to help farmers to gain a comprehensive understanding of pain and guide them to manage pain effectively.

Access to pain education resources is crucial for managing persistent pain, particularly among farmers who generally prefer self-managing their condition [47]. However, most existing pain education resources are primarily designed for the general population rather than tailored explicitly to farmers [20,47,48]. Studies show that farmers tend to prefer educational resources in straightforward, farmer-friendly language, with visuals that are also culturally relevant and appropriate [23,33]. Farmers are likely to be discouraged from utilising health education resources if they may perceive the resources lack an understanding of their specific backgrounds and cultural contexts [49,50,51]. Farmers also often trust their peers who understand their background. Therefore, involving farmers in the development of this resource is likely to be beneficial in helping to overcome the barriers of translating health information to farmers [52,53]. Involvement in co-design can foster the perception that their trusted peers endorse the resource [54], which is especially pertinent given that Australian farmers often place greater trust in peer relationships than in professionals [14]. Such engagement enhances farmers’ sense of belonging to the resource [55], which may lead to increased acceptance, commitment, and sustained engagement [56]. There are limited resources and initiatives that have utilised participatory co-design methods to develop content specifically for farming populations, but these tend to focus on general health promotion, ergonomic practices, and/or pain education and management specific to back pain [23,28,57,58]. Our resource is the first to incorporate such co-design methods and efforts in a persistent pain management education resource targeting farming communities.

The involvement of pain educators, researchers and pain clinicians in our co-design process may further strengthen the wider acceptability of the resource, not only among farmers but also among pain clinicians. As pain clinicians may not accept a resource unless the content is based on research evidence and safe to practice [59], we also ensured that the content is based on research evidence and discussed the clinical safety of the resource’s content with the pain clinicians. Co-researchers emphasised that their GPs and physiotherapists are best suited to disseminate pain education information. There is a possibility of partnering with rural pain networks and farm organisations for broader dissemination, as these partnerships can help mitigate barriers to delivering pain education to rural populations [60]. Possible strategies to deliver pain education to farmers may include utilising local media networks to inform farmers, reaching farmers at local events, sharing pain education information with community champions, and using trusted online platforms [9,23,54]. Involving farmers in the development of this resource is helpful in achieving this balance between the application of evidence-based guidelines and the individual needs of farmers [61].

Our co-researchers also emphasised the importance of providing the resource in both online and printable formats [23,62], as farmers have mixed views and preferences to access printed and/or online formats [33,63]. Furthermore, the resource should be clear, concise, and have farmer-friendly language and illustrations to facilitate dissemination and practical application, consistent with findings from past qualitative research asking what pain means to farmers [9]. Our prototype resource is available as a simple web-based document that farmers can download and use when internet connectivity is poor, and it is capable of being used across common mobile and desktop devices. This increases the accessibility of the pain education resource, in the hope that more farmers will be able to use it [64].

Aligning with other farmer-focused research, our co-researchers emphasised the value of diverse demographic representation in imagery of farmers and they strongly suggested avoiding negativity [23,65]. Farming is traditionally predominantly viewed as a male-dominated occupation, with women farmers often receiving limited recognition [66]. However, this disparity should not be reflected in the pain education resource to ensure its acceptability among women farmers [67]. The prototype resource was enhanced through the inclusion of additional illustrations portraying female farmers. Additionally, considering age diversity is essential to enhance the resource’s appeal to both younger and older generations [68]. This was accomplished by including illustrations of various age groups. Incorporating demographic diversity into health education research is often overlooked [69], yet it is crucial for addressing the needs of a culturally sensitive and stoic group that encounters more barriers than the general population for pain management [70].

### 4.1. Strengths and Limitations

The final prototype resource was formally accepted and endorsed by all co-researchers, indicating that the co-design workshop activities and methods employed at each stage of the study were effectively structured and aligned with the needs of Australian farmers (end-users), and also established evidence-based guidelines for persistent pain management. The development of this prototype has been farmer-led from the outset. Our previous qualitative research findings [9] revealed the need for a farmer-focused persistent pain management resource, specifically having a pain management resource available in both online and printable formats, with clear, concise, and farmer-friendly language and illustrations that encourage farmers’ practical application and dissemination. We brought forward this need and their preferences to inform this co-design study.

This study aligns with key goals of the Australian National Strategic Action Plan for Pain Management [59], namely “(1) people living with pain are recognised as a national and public health priority; (2) consumers, their carers and the wider community are more empowered knowledgeable and supported to understand and manage pain; (4) people living with pain have timely access to consumer-centred best practice pain management including self-management, early intervention strategies and interdisciplinary care and support; and (6) knowledge of pain flourishes and is communicated to health practitioners and consumers through a national research strategy”. This is the first of its kind for a persistent pain management education resource developed with and for farmers. We submitted the completed protocol prior to conducting this research to increase the transparency of this work [71].

Limitations of this study include the absence of a formal prototyping workshop stage involving co-researchers, and a more limited number of participants being involved in the co-design process than initially anticipated, due to time and resource constraints. Although our target sample size of up to n = 10 farmers and n = 10 clinicians was not reached (recruited n = 4 farmers, n = 4 clinicians), our aim to conduct small-group workshops that achieved a deep, generative interactive process among the co-researchers was maintained, and the sample size aligns with guidance from the focus group literature [40]. However, we acknowledge that a larger and more diverse sample could have captured a wider range of perspectives from the highly diverse Australian farming population and enhanced data richness in this study. Consequently, selection bias may have influenced our findings—while our sample was diverse in gender, age, location, and types of pain, we were only able to recruit farmers with capacity and willingness to participate in health research—thus, there is a risk that the most critical target audience for this resource may not be adequately represented (e.g., stoic, treatment-resistant farmers). However, co-researchers reported a range of negative experiences with healthcare in relation to their pain during the pain journey mapping task (e.g., inadequate pain education, having to rely heavily on medications, and frustration about limited recovery and looking for alternative options)—these reflections are consistent with our qualitative work with Australian farmers, suggesting that these experiences are likely to be ubiquitous and will generalise to diverse farming populations. Further, due to feasibility constraints, we were not able to conduct a prototyping workshop as planned, which could have enhanced co-researcher involvement and accountability over final prototype format, usability, and content acceptability. As a workaround, the authors ensured farmers and clinicians were afforded ample opportunities to develop and refine the resource format and content, both during the co-design workshops and via independently reviewing the prototype outside of the workshops, and the resource has been iteratively adapted in line with co-researcher feedback. Further, the authors acted in partnership with co-researchers as part of the overall co-design team and contributed their vast expertise in pain education and management to create the content of the resource, with the format directly informed by our qualitative work with independent farmers [9] and workshop 1 findings. The research team adhered to a rigorous methodology, including respondent validation, verification of findings via video-recording, involvement of at least two investigators in independent data translation, utilisation of best practice guidelines for content development of the resource, and adherence to the GRIPP guidelines [37] for reporting.

### 4.2. Future Directions

While effectiveness testing was beyond the scope of the current study, a longitudinal implementation study should formally test the acceptability, utilisation, and clinical effectiveness of this resource in the future. This work should be conducted in a broader, more diverse sample of farmers, using formal purposive sampling and flexible models that are not dependent on capacity and willingness to contribute to health research, to ensure the views of this population are fully canvassed, including critical hard-to-reach segments such as those who are treatment-resistant. Feedback from a wider group of health professionals (e.g., rural GPs) may also aid further refinement of the resource. Additionally, incorporating other online platforms, such as videos and podcasts, could enhance the delivery of pain education to farmers. While this resource is available in both digital and printable forms and is freely accessible to farmers globally, it was developed with and for Australian farmers. Future research is needed to examine the acceptability and cultural appropriateness of this resource in farming populations around the world. However, the use of clear, straightforward, farmer-friendly language and illustrations is widely applicable to other global farming communities [9,65], which supports that this resource has the potential to be readily adapted for farming populations in other parts of the world, particularly those with a similar sociocultural context. Nonetheless, this study provides a foundation for future co-design research focused on persistent pain management within farming communities and offers a framework and findings that can be adapted and applied to the development of other farmer-focused, health-related education materials. Sharing findings such as these is important to avoid unnecessarily over-burdening at-risk populations with participation in work of this nature. Future research could also further explore the value and preferred models of the implementation of pain prevention programs during the early stages of farmers’ careers, as suggested by co-design participants.

## 5. Conclusions

This participatory co-design study underscores the significance of utilising clear, concise, and farmer-friendly language and illustrations in the development of a relevant, relatable and accessible pain education resource tailored for Australian farmers. The resulting resource is grounded in evidence-based guidelines for persistent pain management. Offering the resource in both online and printable formats may enhance its accessibility and promote broader adoption within farming communities. This is the first evidence-based pain management resource tailored to Australian farmers’ preferences and needs. Addressing this poorly managed persistent pain problem in the Australian farming population is important not only for the health and wellbeing of farmers, but also the overall productivity and quality of life of the farming communities.

## Figures and Tables

**Table 1:** Eligibility Criteria.

Farmers	Pain Clinicians
Over 18 years of age, AND Currently OR have recently owned OR played an active role in the operation of a farming OR pastoral enterprise in Australia, AND Currently suffering from persistent (long-lasting/recurring) pain OR have suffered from persistent (long-lasting/recurring) pain within the last 12 months, AND Living in a non-metropolitan area	Over 18 years of age, AND Currently OR have recently worked as a registered professional in a field of pain science OR worked in a field of pain management OR worked in a field of allied health that involves working directly with persistent pain patients, AND Living and working in a non-metropolitan area

**Table 2:** Workshop Activities.

Activity	Description	Purpose and Strategies
Pre-workshop	All co-researchers were informed of the overall structure and objectives of the workshop.	To familiarise the co-researchers with the aims, subject matter, and methodologies to be employed in the workshop.
Introductory presentation	The chief investigator delivered the opening remarks and introductory presentation covering: What chronic/persistent pain is What a pain management resource is Preliminary research findings What co-design is How we have planned the workshops The co-researchers’ role in the workshops Each member of the research team was introduced then asked to share an interesting fact about themselves as an ice-breaking activity. Subsequently, each co-researcher introduced themselves, with encouragement to share an interesting fact.	To facilitate mutual acquaintance and to familiarise participants with each other’s backgrounds, ensuring that the workshop environment remains interactive and conducive to open sharing of experiences. Furthermore, introductory activities aimed to reduce perceived power hierarchies between farmers, clinicians, and the research team.
Role of the co-researchers and the terms of engagement in the workshops	It was emphasised that all participants hold a collaborative partnership role, with equal rights and contributions to the workshops. It was also noted that respectful disagreements are generally accepted. Prior to recording the workshop via Zoom screen capture—both audio and video—verbal consent was obtained from all participants. It was explained that co-researchers could choose whether or not to respond to any questions or activities during the workshop. They were also provided with an online workbook to submit anonymous written responses. Additionally, they were told to take breaks or turn off the camera when necessary.	To promote respectful discourse and encourage active participation, thereby maximising collaboration and partnership. The workbook was designed to provide an opportunity for anonymous/confidential contributions for co-researchers to document ideas they might feel hesitant to share openly during the workshop (e.g., personal experiences or ideas perceived as discordant from the rest of the group).
Prompt questions and activities for Workshop 1: Brainstorming	There were six prompt questions. What are the top 3 most important things farmers would want to achieve using a pain management resource? (Consensus-based activity) What would be the first thing you would search for in a pain management resource for farmers? (Consensus-based activity) What type of formats would you like in the resource? (Second prompt: what types of resources do you/your clients like/dislike to use?) Who would be best to communicate pain management information to farmers? What kind of communication strategies (e.g., language and images) would you want to/not want to be included in the resource? If the most common barriers were removed (access, time, availability, and other barriers), what would be the ideal way for farmers to manage pain? (Consensus-based activity) There was a case scenario about a typical farmer with persistent pain, named ‘John’. There were three prompt questions related to the case scenario (see Table S3 for the case scenario). What would inspire you (‘John’) to access the resource in the first place? What needs to be included in the resource to encourage you (‘John’) to keep using the resource or try out the suggested strategies? What would make you (‘John’) quit the resource?	The primary objective of the first stage of the workshop was to collaboratively brainstorm the format and mode of the pain management resource. Facilitated discussion involved prompting questions using prioritisation, positive/negative framing, and imaginative journeys to identify key needs, stimulate collaborative problem solving, avoid formats/modes that are not culturally appropriate for farmers, and determine the most effective format and delivery modes for pain management. A case scenario of a typical farmer was employed to help co-researchers reflect on shared persistent pain experiences amongst farmers. The case scenario was developed based on previous qualitative research work of the research team. Co-researchers were asked to imagine that they were the farmer in the case scenario (perspective-taking). This approach aimed to encourage farmers to detach themselves from personal situations and minimise sensitivities around sharing personal information/beliefs, to facilitate open discussion about common experiences and speaking on behalf of other farmers.
Pain journey mapping	This was the final interactive activity at the Brainstorming stage. The trajectories of farmer pain experiences from onset through subsequent developments (e.g., management strategies) to the present day were collaboratively captured on a collective ‘master’ map in real-time using the Miro platform by a designated member of the research team. Farmers were invited to share their personal pain journeys first (contingent upon their comfort), detailing onset, previous management, and future plans. The pain clinicians added to the collective map based on their farmer clients’ typical pain trajectories (e.g., common experiences not yet captured on the map). Farmers and pain clinicians were then asked to identify time points on the collective map at which a pain management resource would be most useful for farmers.	To guide the resource content based on critical time points at which the pain management resources would be useful for farmers.
Developing the prototype resource (Prototyping Stage)	The research team developed a prototype resource between the Brainstorming and Refining stages, based on best-practice guidelines, with its format and mode informed by brainstorming workshops. Prompt questions, case scenarios, and collaborative activities were used to refine the prototype, employing consensus-based approaches.	The research team was responsible for determining the evidence-based content of the pain management prototype resource, utilising their expertise in pain education and farmer health research. We solicited input from the co-researchers regarding the presentation style and language/tone of the content. An illustrator with expertise and experience in creating farmer-focused illustrations was recruited to design the imagery, which co-researchers helped refine by providing detailed feedback.
Pre-workshop after the Prototyping Stage	All co-researchers were informed regarding the overall structure and objectives of the workshop. A preliminary prototype of the pain management resource was distributed to all co-researchers two days before the refining workshops. Co-researchers were instructed to review the prototype and note down any comments or observations for discussion during the upcoming session. The findings of the brainstorming workshops, including the pain journey mapping, were succinctly presented at the beginning of the refining workshops. Co-researchers were asked to verify the interpretation of the findings.	To familiarise the co-researchers with the aims, subject matter, and methodologies employed in the workshop. To review the prototype and make remarks before the refining workshops to enable co-researchers to actively participate in and reflect on the subsequent workshop phase. To present previous workshop findings to gain respondent validation in an attempt to make co-researchers feel heard, valued and respected, and to enhance their collaboration towards refining workshops. Additionally, to allow the research team to interpret the findings more accurately. To ensure we accurately capture a farmer’s typical pain journey and that no key time points were missed.
Prompt questions and activities for Workshop 2: Refining	The initial case scenario was updated (given that the typical farmer now has access to the prototype pain management education resource) and accompanied by four prompt questions. Co-researchers were invited to evaluate the pain management prototype as if they were “John” to facilitate discussion around these prompts (see Table S3 for the modified case scenario). What would John’s initial impression be of the new resource? (Consensus-based activity) What would encourage John to use this pain education resource? (Consensus-based activity) What would encourage John to recommend this resource to other farmers? (Consensus-based activity) What would stop John from using this resource? (Consensus-based activity) Finally, the co-researchers were asked for any additional suggestions regarding the content, wording, images, and layout to enhance the resource.	The primary objective of the second stage of the workshop was to gather feedback on the prototype pain management resource and refine it accordingly. Co-researchers were again asked to imagine they were the farmer in a revised case scenario (perspective-taking). The revised case scenario enabled co-researchers to evaluate the credibility, acceptability and feasibility of the prototype resource among farmers, as well as its clinical safety for implementation. It also enabled farmers to detach themselves from personal situations and minimise sensitivities around sharing personal information/beliefs, to facilitate open discussion about common experiences and speaking on behalf of other farmers.

**Table 3:** Co-researcher Information.

	Farmers (n)	Pain Clinicians (n)	Researchers (n)
**Pre-workshop (Pilot Testing)**	Male = 1 Female = 1	N/A	Male = 1 Female = 3
**Workshop 1 (Brainstorming Stage)**	Male = 3 Female = 1	Male = 1 Female = 3	Male = 1 Female = 1
**Prototyping Stage**	N/A	N/A	Male = 2 Female = 3
**Workshop 2 (Refining Stage)**	Male = 2 Female = 1	Female = 1	Male = 1 Female = 2
**Written responses from co-researchers unable to attend the Refining Stage workshop**	Male = 1	Female = 1	N/A

**Table 4:** Key suggestions for the prototype during Workshop Stage 2 (refining stage).

Area	Suggestion	Action Taken
Farmer illustrations	Most farmer illustrations depict older male farmers and are not representative enough of the diversity in the farming population. Include another female farmer in the illustrations.	Redesigned the female illustrations to make the female image more feminine. Changed the ‘male farmer stretching’ to a female. Redesigned male farmers’ illustrations to represent different age groups.
Diagnosis before taking action	It is highly recommended to see a healthcare professional and rule out any serious underlying health conditions before trying self-management strategies.	Added a statement on the prototype resource, “When anyone has chronic pain, it is important to consult your healthcare professional to rule out any unusual illness that might be contributing.”
Quotes from farmers’ lived experience	Keep the quotes in first person. Provide more context for the quotes, such as age and name. Remove any sensitive words (e.g., ‘survive’) from the quotes.	Added new quotes from farmers’ lived experience, and presented quotes with further demographic details (name, age, and gender). Ensured no sensitive words are included in the quotes.

## Data Availability

The deidentified data associated with this manuscript are available from the corresponding authors upon reasonable request.

## Supplementary Materials

The following supporting information can be downloaded at: https://www.mdpi.com/article/10.3390/ijerph23081015/s1, Table S1: Deviations from the original protocol; Table S2: Pilot Workshop—Suggestions and Feedback; Table S3: Case scenario activities; Table S4: Complete list of actions and suggestions from the workshop stage two for the prototype draft; Table S5: Example Quotes; Table S6: GRIPP 2 Short Form.
