# Supplementary material for: Co-Designing a Consumer-Centred Educational Resource to Assist Australian Farmers with the Management of Persistent Pain
Source: Int J Environ Res Public Health. 2026 Aug 3;23(8):1015. doi: 10.3390/ijerph23081015 (PMC13513496; doi:10.3390/ijerph23081015)
Supplement: Supplementary file 1 [file ijerph-23-01015-s001.zip › ijerph-4435278-supplementary.pdf]

Supplementary Table S1: Deviations from the original protocol
(Original Protocol: https://osf.io/5zeuq/overview: accessed on 25 August 2025)

Item	Description
Sample size	The original plan was to recruit 10 farmers and 10 clinicians for the study; however, only 4 farmers and 4 clinicians were included in this study.
Workshop Stage Two (prototyping)	The original plan was to conduct a formal prototyping stage with all co-researchers; however, due to time and resource constraints, we employed the expertise within the research team to develop the content of the prototype pain education resource.

Supplementary Table S2: Pilot Workshop- Suggestions and Feedback

Suggestions and Feedback
Introduction presentation: Need more information about what a pain management resource would like at the outset
Prompt question 1: a very direct and difficult question to start with. Wording should be clearer and simpler. Give examples.
Prompt question 4: confusing wording for farmers. Need to simplify. Remove word "hate"
Consensus-seeking activities: reframe the activity to have the co-researchers rank/ prioritise their answers
Pain journey activity: keep the explanation simple. Let co-researchers tell their stories while one team member works on the Miro board.
Build multiple pain journeys on one Miro board. Could get them to map pains and treatments, then, as a whole group, reflect on when pain education is needed.
Prompt questions 3,5,6: Good discussions.
Case scenario: if there is any co-researcher called 'John', ensure the case scenario name is changed.

Supplementary Table S3: Case scenario activities

Case Scenario	Questions
**Brainstorming Stage**	
• 45-year-old farmer from rural Australia<br>• Married to Jane and has three kids.<br>• Chronic shoulder and back pain for more than 7 years.<br>• Every morning, he struggles to get out of bed due to his back pain.<br>• Committed to the farm but starts slow because of pain.<br>• Does not have anyone to help him with the farm except Jane.<br>• Feels tired, exhausted and in pain as the day progresses.<br>• Pain disturbs his sleep.<br>• The only thing John does for his pain is take some painkillers.<br>• Jane is worried that John is getting physically unfit.<br>• Does not like to go to his GP or other health professionals.	1. What would inspire you (John) to access the resource in the first place?<br>2. What needs to be included in the resource to encourage you (John) to keep using the resource or try out the suggested strategies?<br>3. What would make you (John) quit the resource?
**Refining Stage**	
• 45-year-old farmer from rural Australia<br>• Married to Jane and has three kids.<br>• Chronic shoulder and back pain for more than 7 years.<br>• Every morning, he struggles to get out of bed due to his back pain.<br>• Committed to the farm but starts slowly because of pain.<br>• Feels tired, exhausted and in pain as the day progresses.<br>• Pain disturbs his sleep.<br>• The only thing John does for his pain is take some painkillers.<br>• John's GP ruled out any underlying conditions and recommended pain education and self-management.<br>• When John searched on the internet about managing chronic pain, he found a number of resources. But he gave up reading those.<br>• He then discovered a new resource specifically designed for farmers.	1. What would John's initial impression be of the new resource?<br>2. What would encourage John to use this pain education resource?<br>3. What would encourage John to recommend this resource to other farmers?<br>4. What would stop John from using this resource?

Supplementary Table S4: Complete list of actions and suggestions from the workshop-stage two for the prototype draft

Suggestion	Outcome	Changes made	Notes
Image 2: Change the big book of pain management guide to a pamphlet	Changes made	Convert the pain management book into a pamphlet.	
Knowledge is power: Remove repetition of the statement	Changes made	Removed the first sentence in this section as per the suggestion.	
Need to diagnose first/ go to a GP	Changes made	Added the below statement.<br>*"When anyone has chronic pain, it is important to consult your healthcare professional to rule out any unusual illness that might be contributing."*	
Moving 'despite pain'- should be guided by a health professional, otherwise risky	Changes made	Removed 'despite pain'. Replaced 'even if it hurts' with *"guided by your health professional."*	
Quotes: "*You gotta go something*", need to keep quotes to first person	Changes made	Removed the old quotes and replaced them with new ones.	
Female illustration: need to make this more feminine (i.e., dark long hair, breasts)	Changes made	Added more feminine features to female illustrations	
Nothing is 100% so change the words "will give you a better life" to "likely to give you a better life"	Changes made	Amended the sentence to *"A counsellor or psychologist can help you manage better with many things that are known to also increase pain, for example stress, anxiety, tricky relationships and low mood. They may also be able to help you alter how your brain processes pain, which in turn can help reduce stress and anxiety, improve sleep, and guide you towards meaningful relationships, which will likely give you a better quality of life."*	
Add a statement for "taking it slowly and going step by step": it's going to be ongoing for the rest of the life, it's	Changes made	Added a statement *"You need to do enough different activities and movements to re-train your pain system to be less overprotective, but not so much activity*	

not going to be quick for everyone, and it's not going to be easy, either. So setting up those expectations from the start		*that it really sets off your warning system."*	
Change the word "strategies" to something lighter	Changes made	Amended the title as *"Below are some other approaches that may help you to manage your ongoing pain."*<br><br>Amended the statements below;<br><br>*"Speak to your GP who can rule out any unusual conditions and help you navigate a way forward. Ask them what they can recommend and what you can do."*<br><br>'There is a close relationship between stress and pain, so, talking to a psychologist or counsellor is another helpful approach to consider'.<br><br>*"They may also be able to help you alter how your brain processes pain, reduce stress and anxiety, improve sleep, and guide you towards meaningful relationships, which will likely give you a better quality of life."*	
Remove the quote "thoughts are just thoughts" or give more context	Changes made	Removed the old quotes and replaced them with new ones.	
Distinguish strategies to self-manage, and therapies etc.	Changes made	Categorised resources as "learn, do, connect."	
Change "have a yarn with someone you trust" to "Have a yarn with a mate" or something like that, or you feel comfortable with, or good farmers ask for help, or ask a mate their opinion	Changes made	Changed the statement to *"Have yarn with a mate."*	
Add playing/ sport/ doing a job or something like that as a strategy	Changes made		Added 'playing a sport' as an example for the first question in

			the ‘reflection and goal setting’ section.<br><br>However, we cannot add as a pain management strategy due to lack of research evidence.
Number of resources depending on the reader	Changes not made		No changes required.
Categorise resources	Changes made	Categorise the Additional information section into 1) Learn, 2) Do, and 3) Connect	
Add more context to quotes, such as age, name	Changes made	Removed the old quotes and replaced them with new ones.	
Lots of farmer stuff is older, male, farmer-focused, and not representative.	Changes made	Illustrations modified for wider demographic representation.	
Focus on other MSK pain, include information on pelvic pain – e.g., further resources. Broader relevance.	Changes made	Added a resource to the ‘Additional Information’ section	
Change the doctor to female, include a female farmer in more scenarios. Female stretching.	Changes made	Changed the male farmer stretching to a female farmer stretching.	
‘Survive’ – change this wording due to current issues with suicide in this population – ‘not going to thrive’ instead? More positive.	Changes made	Added new farmer quotes.	
Page 3 (bottom right) – practicing positive thinking and healthy diet – may be patronising.	Changes made	Changed the section to ‘explore mindfulness’, and replaced ‘positive thinking’ with<br>‘Learn some ways to cope, like being aware of emotions and how these can impact pain’.	

Can be dismissive in chronic illness ('all in head'), quite often dismissed by health professionals – change the language e.g., be aware of emotions and how these can impact pain.	Changes made	Amended the statement as "Learn some ways to cope, like being aware of emotions and how these can impact pain".	
Change healthy diet to an anti-inflammatory diet – healthy diet too broad/vague, expand on this, examples of foods. Learn how your diet can impact your pain. Information on what is actually pro- and anti-inflammatory foods.	Changes made	Expanded this section as "Consider adopting a healthy diet, including anti-inflammatory foods like fruits, vegetables, and healthy fats".	
Resource in written format – need to emphasise importance of clearing red flags/underlying health condition, working with a health professional before trying out the recommended strategies – qualification that this is for people who have been checked by a health professional.	Changes made	Added a statement on page 1 as outlined below.<br><br>"It is strongly advised to consult your healthcare professional to rule out any underlying illness causing your pain".<br><br>Sweet Zone – it is already mentioned that they may need to get help from a professional ('Finding your sweet spot can be tricky, but with time and patience (and perhaps some help from a professional), you will be able to do this')	
Fact sheets with resources – hyperlinks useful for online documents, but not helpful for printed handout – make the websites easy to find (add website name)	Changes not made		No changes required
Reflection and goal setting – expand on this, more blank templates – interesting and helpful – getting it down onto paper, proactive.<br><br>Goal setting: think about common excuses – haven't got time, costs	Changes made	Re-structured the section	

money to see a practitioner – use these as hooks, cost benefit of using these strategies – do jobs			
Stress and pain – expand on this relationship, explore the correlation in their life. Link to healthy eating and mindfulness. Stress might be a factor preventing recovery.<br><br>Stress and things you can control, self-efficacy – filter this through in the wording – things you can do to control your pain, stress reducing, boosting ability to control certain things. May control/easier to control – use this language instead of 'can' – many farmers are control freaks, but there is also a lot you can't control e.g., weather	Changes made	Added a statement as outlined below.<br><br>"They may also be able to help you alter how your brain processes pain, reduce stress and anxiety, improve sleep, and guide you towards meaningful relationships, which will likely give you a better quality of life".	
Circle of control – things you can and can't control<br>Diet, physical activity, things you can control	Changes made	Modified the section.	
Font size – currently 12pt, no smaller	Changes not made		No changes required

Supplementary Table S5: Example Quotes

Area	Suggestion	Quotes
Farmer illustrations	Most farmer illustrations depict older male farmers and are not representative. Include another female farmer in the illustrations.	*"If I looked at this, I'd just instantly think male, kind of, you know, 50-year-old male, shoulder injury, knee injury, hip injury, back, that kind of stuff. So it wouldn't resonate with me."* (Farmer KB, female)<br><br>*"I would like to see a female farmer, because I think if you've got the male farmer in every scenario, you're only appealing to male farmers. ...And it's a bit kind of, stereotypical to have a female health practitioner and a male."* (Farmer KB, female)
Diagnosis before taking action	It is highly recommended to see a healthcare professional and rule out any serious underlying health conditions before trying self-management strategies.	*"So if they don't know what the problem is, they may go to the GP first and try to get an overall diagnosis, before they go to a remedy. If it's something caused by an illness, such as the cancer, pain, or something like that. Who knows?"* (Farmer MC, male)<br><br>*"I think that that is often what drives people to seek healthcare for pain, is that fear that it might be something big, it might be something bad."* (Clinician MA, female)
Quotes from farmers' lived experience	Keep the quotes in first person. Provide more context for the quotes, such as age and name. Remove any sensitive words (e.g., 'survive') from the quotes.	*"There's a farmer there saying, 'you've gotta do something different'. Now, that's someone telling me, I've got to do it rather than saying, like most farmers, I used to work through the pain or whatever. Keep it in first person."* (Farmer MC, male)<br><br>*"The language there, I know you can't change it because it's a quote, but I don't like the 'survive' thing, just because of the things that are happening at the moment, there is a lot of talk of suicide."* (Farmer KB, female)

Supplementary Table S6: GRIPP 2 Short Form

Section	Item	Reported on the page number
Aim	Report the aim of PPI in the study	3 of 21
Methods	Provide a clear description of the methods used for PPI in the study	3—9 of 21
Study results	Outcomes—Report the results of PPI in the study, including both positive and negative outcomes	9—13 of 21
Discussion and conclusions	Outcomes—Comment on the extent to which PPI influenced the study overall. Describe positive and negative effects	13—17 of 21
Reflections/ critical perspective	Comment critically on the study, reflecting on the things that went well and those that did not, so others can learn from this experience	15—16 of 21
